# Fabrication of Core Crosslinked Polymeric Micelles as Nanocarriers for Doxorubicin Delivery: Self-Assembly, *In Situ* Diselenide Metathesis and Redox-Responsive Drug Release

**DOI:** 10.3390/pharmaceutics12060580

**Published:** 2020-06-23

**Authors:** Yihenew Simegniew Birhan, Haile Fentahun Darge, Endiries Yibru Hanurry, Abegaz Tizazu Andrgie, Tefera Worku Mekonnen, Hsiao-Ying Chou, Juin-Yih Lai, Hsieh-Chih Tsai

**Affiliations:** 1 Graduate Institute of Applied Science and Technology, National Taiwan University of Science and Technology, Taipei 106, Taiwan; yihenews@gmail.com (Y.S.B.); fentahunhailebdu@gmail.com (H.F.D.); idrisbrhm7@gmail.com (E.Y.H.); habegaz21@gmail.com (A.T.A.); tefe16@gmail.com (T.W.M.); wherelove8@gmail.com (H.-Y.C.); jylai@mail.ntust.edu.tw (J.-Y.L.); 2 Advanced Membrane Materials Center, National Taiwan University of Science and Technology, Taipei 106, Taiwan; 3 R&D Center for Membrane Technology, Chung Yuan Christian University, Chungli, Taoyuan 320, Taiwan

**Keywords:** core crosslinked micelles, diselenide bond, redox-responsive, drug delivery

## Abstract

Polymeric micelles (PMs) have been used to improve the poor aqueous solubility, slow absorption and non-selective biodistribution of chemotherapeutic agents (CAs), albeit, they suffer from disassembly and premature release of payloads in the bloodstream. To alleviate the thermodynamic instability of PMs, different core crosslinking approaches were employed. Herein, we synthesized the poly(ethylene oxide)-*b*-poly((2-aminoethyl)diselanyl)ethyl l-aspartamide)-*b*-polycaprolactone (mPEG-P(LA-DSeDEA)-PCL) copolymer which self-assembled into monodispersed nanoscale, 156.57 ± 4.42 nm, core crosslinked micelles (CCMs) through visible light-induced diselenide metathesis reaction between the pendant selenocystamine moieties. The CCMs demonstrated desirable doxorubicin (DOX)-loading content (7.31%) and encapsulation efficiency (42.73%). Both blank and DOX-loaded CCMs (DOX@CCMs) established appreciable colloidal stability in the presence of bovine serum albumin (BSA). The DOX@CCMs showed redox-responsive drug releasing behavior when treated with 5 and 10 mM reduced glutathione (GSH) and 0.1% H_2_O_2_. Unlike the DOX-loaded non-crosslinked micelles (DOX@NCMs) which exhibited initial burst release, DOX@CCMs demonstrated a sustained release profile in vitro where 71.7% of the encapsulated DOX was released within 72 h. In addition, the in vitro fluorescent microscope images and flow cytometry analysis confirmed the efficient cellular internalization of DOX@CCMs. The in vitro cytotoxicity test on HaCaT, MDCK, and HeLa cell lines reiterated the cytocompatibility (≥82% cell viability) of the mPEG-P(LA-DSeDEA)-PCL copolymer and DOX@CCMs selectively inhibit the viabilities of 48.85% of HeLa cells as compared to 15.75% of HaCaT and 7.85% of MDCK cells at a maximum dose of 10 µg/mL. Overall, all these appealing attributes make CCMs desirable as nanocarriers for the delivery and controlled release of DOX in tumor cells.

## 1. Introduction

The recent advancements in synthetic chemistry and nanotechnology fostered the development of different nanocarriers for the delivery and controlled release of chemotherapeutic agents (CAs) in tumor cells [1,2,3,4,5,6]. Polymeric micelles (PMs), characterized by small size, appreciable drug-loading content (DLC), better accumulation in tumor tissue via “enhanced permeation and retention (EPR) effect”, and the ability to avoid detection and subsequent clearance by the mononuclear phagocyte (MNP) system, are convenient to improve the poor aqueous solubility, slow absorption and non-selective biodistribution of payloads and hence, enhance the therapeutic efficacy of CAs [7,8,9,10,11,12]. Despite the aforementioned appealing attributes of PMs as carriers of CAs, only a few micellar formulations advance beyond the preclinical development stage [13]. This may be attributed to the poor thermodynamic stability of PMs due to their non-specific interaction with blood protein components, and failure to withstand a subclassification of compressive, tensile, and shear stresses in vivo which leads to premature release of their cargo and non-specific biodistribution upon intravenous administration [14,15,16,17]. To circumvent the in vivo instability of PMs, covalent crosslinking of the micellar shell or core were often employed [10]. As the former suffered from aggregation, reduced stealthiness and weakened EPR effect [18,19,20,21], core crosslinking has attracted considerable attention in the fabrication of stable PMs intended for anticancer drug delivery applications (DDAs) [22,23,24]. To achieve the on-demand release of CAs in the region of interest, stimuli-responsive core crosslinking agents with dynamic covalent bonds (DCBs) are in high demand [10,12,15,25]. In this regard, S–S and Se–Se bonds containing PMs [23,26,27] have shown preferential sensitivity towards tumor tissue redox signals (2–10 mM reduced glutathione (GSH) and 1 mM H_2_O_2_) over extracellular cancer environments (2–10 µM reduced GSH and 20 nM H_2_O_2_) [17,28,29]. Thus, dynamic S–S and Se–Se bonds containing crosslinking agents are ideal to maintain the in vivo stability of PMs and achieve controlled release of payloads in the milieu of cancer tissue. 

The lower Se–Se bond energy, larger atomic size, and weak electronegativity of selenium endowed Se–Se bonds with a more dynamic nature and higher redox responsiveness than S–S bonds [30,31]. Moreover, the induction of diselenide metathesis reactions in the presence of visible light or mild heat (˂100 °C) as compared to disulfide exchange reactions that demand catalysts and/or harmful UV irradiation [32], inspired researchers to engineer Se–Se bonds containing shell or core crosslinked micelles (CCMs). For instance, Zhai et al. prepared visible light-induced CCMs from a PEG-*b*-PBSe diblock polymer. The diselenide-bearing CCMs demonstrated reasonable stability in physiological conditions with appreciable redox-responsive camptothecin (CPT) and doxorubicin (DOX) release in tumor tissue [23]. Indocyanine green (ICG) and DOX-loaded CCMs (DOX@CCMs) were prepared by a [4+2] cycloaddition reaction between PEO-*b*-PFMA and diselenide containing a bismaleimide crosslinker [33]. The micelles exhibited near-infrared (NIR) triggered Se–Se bond cleavage and rapid release of DOX, leading to enhanced apoptosis in HepG2 cells. Deepagan et al., prepared in situ crosslinked, physiologically stable PMs which showed a rapid release of DOX in the reactive oxygen species (ROS) rich environment. Because of their thermodynamic stability, the DOX-loaded micelles delivered significantly more drugs to tumor tissue after systemic administration into tumor-bearing mice [28]. Although Se-Se-bearing shells or CCMs presented extra serum stability and tumor tissue accumulation in vivo models, empirical data are still scarce. Thus, fabrication of diselenide-linked, redox stimuli-responsive CCMs as anticancer drug carriers is in utmost demand.

**Scheme 1:**
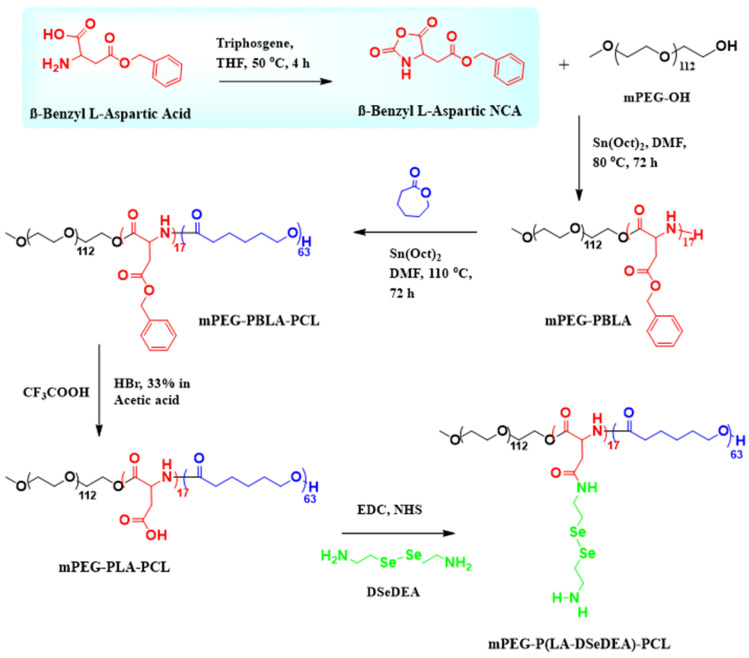
Synthetic routes of the redox-responsive mPEG-P(LA-DSeDEA)-PCL copolymer.

Herein, we synthesized the poly(ethylene oxide)-*b*-poly((2-aminoethyl)diselanyl)ethyl l-aspartamide)-*b*-polycaprolactone (mPEG-P(LA-DSeDEA)-PCL) copolymer (Scheme 1) with pendant selenocystamine (Sec) groups by employing the ring-opening polymerization (ROP), hydrolysis, and EDC/NHS coupling reactions. The amphiphilic copolymer self-assembled into stable CCMs owing to visible light-induced diselenide metathesis reactions between the Sec moieties [23,25,34]. In this copolymer architecture, the biocompatible and FDA approved hydrophilic mPEG segment would form the shell of the micelles and help to avoid the non-specific interactions between serum proteins, cellular structures, and the micelles surface. Its “stealth” property also helps to avoid detection and subsequent clearance by the MNP system from the body [35], resulting in a sustained plasma concentration and selective accumulation into tumor tissue via the EPR effect [36]. CCMs comprise highly biocompatible, biodegradable and non-toxic polyesters and polypeptides, and are ideal candidates for DDAs [20,37]. Polyesters and polypeptides can be easily synthesized by ROP reaction to give well defined products. Moreover, the functional groups of polypeptides can be chemically modified to fine-tune the stability, drug encapsulation, and release profiles of PMs [38]. To this end, we incorporated poly((2-aminoethyl)diselanyl)ethyl l-aspartamide) (P(LA-DSeDEA)) and PCL blocks that could constitute the micellar corona and harbor the poorly soluble anticancer drug, DOX. Then, the effect of diselenide exchange triggered crosslinking on hydrodynamic diameter (*D*_h_), DLC, and colloidal stability were systematically investigated. In addition, the in vitro drug-releasing trend of DOX@CCMs was investigated in the presence of reduced GSH (5 and 10 mM) and 0.1% H_2_O_2_ and compared with their precursor, DOX-loaded non-crosslinked micelles (DOX@NCMs). The in vitro cytotoxicity of DOX@NCMs and DOX@CCMs was examined using the MTT assay in HaCaT, MDCK and HeLa cell lines. Moreover, the cellular internalization of DOX@NCMs and DOX@CCMs were also analyzed using a fluorescent microscope and flow cytometry.

## 2. Materials and Methods

### 2.1. Materials

Methoxy poly(ethylene oxide) with an average molecular weight of ~5000 g/mol, l-aspartic acid β-benzyl ester, triphosgene (98%), ε-caprolactone (97%), stannous 2-ethylhexanoate (Sn(Oct)_2_, 92.5–100%), *N*-hydroxysuccinimide (NHS), 1-ethyl-3-(3-dimethylaminopropyl)carbodiimide (EDC), anhydrous *N*,*N*-dimethylformamide (DMF, 99.8%), dichloromethane (DCM, 99.8%), dimethylsulfoxide (DMSO), anhydrous tetrahydrofuran (THF, 99.9%), pyrene (98%), Rhodamine B (RhB), triethylamine (TEA, 99.5%), anhydrous diethyl ether, paraformaldehyde (97%), anhydrous hexane (99.5%), doxorubicin hydrochloride (DOX·HCl), trifluoroacetic acid (TFA), and hydrobromic acid (in 33 wt.% acetic acid), were products of Sigma Aldrich. Additionally, 4’,6-diamino-2-phenylindole (DAPI), Dulbecco’s modified Eagle medium (DMEM), 3-[4,5-dimethylthiazol-2-yl]-2,5-diphenyltetrazolium bromide (MTT), fetal bovine serum (FBS), penicillin-streptomycin, trypsin, and Dulbecco’s phosphate-buffered saline (PBS) were obtained from GIBCO Invitrogen Corp. Selenocystamine dihydrochloride was purchased from Combi-Blocks, San Diego, CA, USA. Unless otherwise specified, reagents were used as received without further purification.

### 2.2. Methods

#### 2.2.1. Synthesis of β-Benzyl-l-Aspartate *N*-Carboxy Anhydride (BLA-NCA)

The synthesis of mPEG-P(LA-DSeDEA)-PCL with pendant Sec moiety was conducted by sequential ROP reaction of mPEG–OH with BLA-NCA and ε-caprolactone followed by hydrolysis of the benzyl ester group and subsequent coupling with selenocystamine dihydrochloride (DSeDEA) through EDC/NHS click chemistry (Scheme 1). The monomer, (BLA-NCA), was synthesized by the cyclization reaction between l-aspartic acid β-benzyl ester and triphosgene, as described in the Fuchs–Farthing method [39]. Briefly, l-aspartic acid β-benzyl ester (1 g, 4.5 mmol) was added to 15 mL of anhydrous THF. Then, 0.8 g (2.7 mmol) of triphosgene was added to the above suspension, heated at 50 °C in an oil bath and stirred in a nitrogen atmosphere for about 4 h. The resulting colorless mixture was concentrated (until 25% of the original volume remained), poured into excess cold anhydrous hexane and kept overnight at −20 °C. Finally, the precipitate was collected, dried under vacuum for 48 h, and stored at 4–10 °C until used for further reactions.

#### 2.2.2. Synthesis of Poly(ethylene oxide)-*b*-Poly(β-Benzyl-l-Aspartate) (mPEG-PBLA)

The diblock copolymer, mPEG-PBLA was obtained by ROP reaction of BLA-NCA wherein mPEG-OH served as a macro-initiator [40]. In brief, mPEG-OH (1 g, 0.2 mmol) was transferred to a three-neck round bottom flask and heated under vacuum at 110 °C to remove the residual moisture. Then, the mixture was cooled to 80 °C and complexed with 0.5 wt.% Sn(Oct)_2_ for 24 h in a nitrogen atmosphere. Next, (1 g, 4 mmol) BLA-NCA was added to the reaction mixture, and 4 mL of anhydrous DMF was injected (to ensure complete dissolution of BLA-NCA) via syringe, stirred and heated at 80 °C for an additional 48 h. After completion of the reaction, the solution was poured into cold diethyl ether and kept at −20 °C for 24 h. In the end, the resulting precipitate was filtered, washed three times with cold diethyl ether (to ensure maximum purity), dried under vacuum for 48 h, and kept at 10 °C until used in subsequent reactions.

#### 2.2.3. Synthesis of Poly(ethylene oxide)-*b*-Poly(β-Benzyl-l-Aspartate)-*b*-Polycaprolactone (mPEG-PBLA-PCL)

mPEG-PBLA-PCL was synthesized by ROP reaction of ε-caprolactone initiated by the terminal amino group of mPEG-PBLA [40]. Firstly, mPEG-PBLA (1 g. 0.12 mmol) was transferred to a three-neck round bottom flask and heated under vacuum at 110 °C to remove residual moisture. Next, 0.5 wt.% Sn(Oct)_2_ and 5 mL anhydrous DMF was added to the mixture and stirred for 24 h in a nitrogen atmosphere. Then, 0.82 g (7.2 mmol) of ε-caprolactone was syringed into the reaction vessel and heated at 110 °C for an additional 48 h while maintaining the neutral reaction environment. Finally, the solution was precipitated in cold diethyl ether and kept at −20 °C for 24 h. The resulting mPEG-PBLA-PCL precipitate was filtered, washed three times via cold diethyl ether, dried under vacuum, and stored at 10 °C for further characterizations and use.

#### 2.2.4. Synthesis of Poly(ethylene oxide)-*b*-Poly(l-Aspartic Acid)-*b*-Polycaprolactone (mPEG-PLA-PCL)

Acid-catalyzed hydrolysis of mPEG-PBLA-PCL resulted in cleavage of the benzyl ester group of the polypeptide block. For this purpose, 0.5 g (0.034 mmol) of mPEG-PBLA-PCL was dissolved in TFA (1 equivalent) and stirred vigorously. After 30 min, 2 equivalents of hydrobromic acid solution (33 wt.% in acetic acid) was added and stirred for 1 h at 50 °C. Finally, the target copolymer was obtained upon precipitation in cold diethyl ether solution [41]. The white precipitate was filtered, washed three times with cold diethyl ether, and dried under vacuum for 48 h and stored at 10 °C.

#### 2.2.5. Synthesis of mPEG-P(LA-DSeDEA)-PCL

The target amphiphilic copolymer with pendant Sec moiety, mPEG-P(LA-DSeDEA)-PCL, was synthesized by grafting mPEG-PLA-PCL with DSeDEA in the presence of EDC and NHS as coupling agents. In brief, 0.3 mg (2.61 mmol per aspartic acid moiety) of mPEG-PLA-PCL and 0.5 g (2.61 mmol) of EDC were added into a three-neck round bottom flask. Then, 20 mL DMF and DMSO co-solvent (3:1 *v*/*v* ratio) was added to the mixture and stirred at room temperature for 30 min. Then, NHS (0.6 g, 5.22 mmol) was added into the reaction mixture and stirred for 6 h for complete activation of the carboxyl group of the PLA segment. Finally, 0.63 g (2.61 mmol) of DSeDEA was added into the reaction mixture and stirred for 48 h at room temperature in a neutral environment. The mixture was poured into large excess cold diethyl ether and the precipitate was filtered, dried, re-dissolved in DMF, and dialyzed against distilled water under visible light protection by using a cellulose dialysis membrane (MWCO 6000–8000 Da) for 48 h. The resulting solution was freeze-dried, and the target product was collected and stored at 10 °C.

#### 2.2.6. Characterization

^1^H NMR and ^13^C NMR spectra were recorded using a Varian Unity-600 NMR spectrometer (Varian, Inc., Palo Alto, CA, USA) at 600 MHz to verify the chemical structures of the synthesized compounds. Fourier transform infrared (FTIR) spectroscopy (PerkinElmer, Buckinghamshire, UK) was used to determine the stretching and bending vibrations of the different functional groups present in the synthesized compounds. Likewise, the characteristic Raman peaks of Se–Se, Se–C, and other prominent functional groups in mPEG-P(LA-DSeDEA)-PCL were investigated by placing a small amount of the sample in a silicon sample holder and the Raman spectra were scanned between 150 and 4000 cm^−1^ using a JASCO NSR-5100 Laser Raman spectrometer. An Advanced Polymer Chromatogram (APC) system with a THF column was used to determine the molecular weights and polydispersities of 2 mg/mL samples (where THF served as eluent) at 45 °C via ACQUITY Advanced Polymer Chromatography (APC™) with a flow rate of 0.8 mL/min. Polystyrene was used as standard for molecular weight calibration. The *D*_h_, ζ-potential of the micelles was determined by using a dynamic light scattering (DLS) instrument with triplicate measurements, and the results were expressed as mean ± SD. The morphology of the CCMs was assessed from the images taken using a field emission scanning electron microscope (FESEM) (JSM-6500F, JEOL). In addition, a UV-vis spectrophotometer (JASCO V-730) and a fluorescence spectrophotometer (JASCO V-330) were used to determine the absorbance and emission spectra of the samples, respectively.

#### 2.2.7. Preparation of mPEG-P(LA-DSeDEA)-PCL Micelles

NCMs and CCMs of mPEG-P(LA-DSeDEA)-PCL origin were prepared by the nanoprecipitation method [23,28]. The mPEG-P(LA-DSeDEA)-PCL copolymer (20 mg) was dissolved in 3 mL of acetonitrile and DMSO co-solvent (2:1 *v*/*v* ratio), 1 mL of water was added dropwise and probe sonicated for 2 min. Then, the mixture was added to 15 mL of water dropwise and stirred vigorously overnight. The acetonitrile and DMSO were removed by dialyzing the mixture against deionized water (MWCO 6000–8000 Da) in a dark environment for 36 h to afford NCMs. CCMs were prepared in the same manner as the NCMs where visible light was irradiated from an ordinary tungsten lamp (25 W) without a filter during the final 2 h of the dialysis process at room temperature to trigger diselenide exchange reaction between pendant selenocystamine groups of mPEG-P(LA-DSeDEA)-PCL chains in the interface between the hydrophobic and hydrophilic regions of the NCMs [42]. The resulting CCMs and NCMs solutions were stored at 10 °C for further use and characterization.

#### 2.2.8. Determination of Critical Micelle Concentration (CMC)

The CMC of mPEG-P(LA-DSeDEA)-PCL was determined by the pyrene fluorescent probe method [43,44,45]. 1 × 10^−3^, 5 × 10^−4^, 1 × 10^−4^, 5 × 10^−5^, 1 × 10^−5^, 5 × 10^−6^, 1 × 10^−6^, 5 × 10^−7^, 1 × 10^−7^, and 5 × 10^−8^ g/mL micellar solutions were prepared by diluting the stoke solution with redistilled water. 2 × 10^−4^ M of pyrene solution in acetone was prepared and 100 µL was subsequently transferred into a series of amber glass vials, and the acetone was evaporated under dark conditions. The copolymer solutions with different serial concentrations were added into the respective vials to give a final pyrene concentration of 2 × 10^−6^ M. All the solutions were sonicated and equilibrated in a water bath for 1 h. The samples were then kept at room temperature for 24 h and the emission spectra of the solutions were measured from 350 to 440 nm by using a fluorescence spectrophotometer with an excitation wavelength of 336 nm and the slit width for both excitation and emission were 2.5 nm. The intensity ratio of the first peak and the third peak (I372/I383) in the pyrene emission spectra were plotted as a function of polymer concentration (log C). Finally, the CMC value of mPEG-P(LA-DSeDEA)-PCL was quantified from the intersection of the tangent to the curve at the inflection with the horizontal tangent through the points of low concentration.

#### 2.2.9. Preparation of DOX-loaded mPEG-P(LA-DSeDEA)-PCL Micelles

**Scheme 2:**
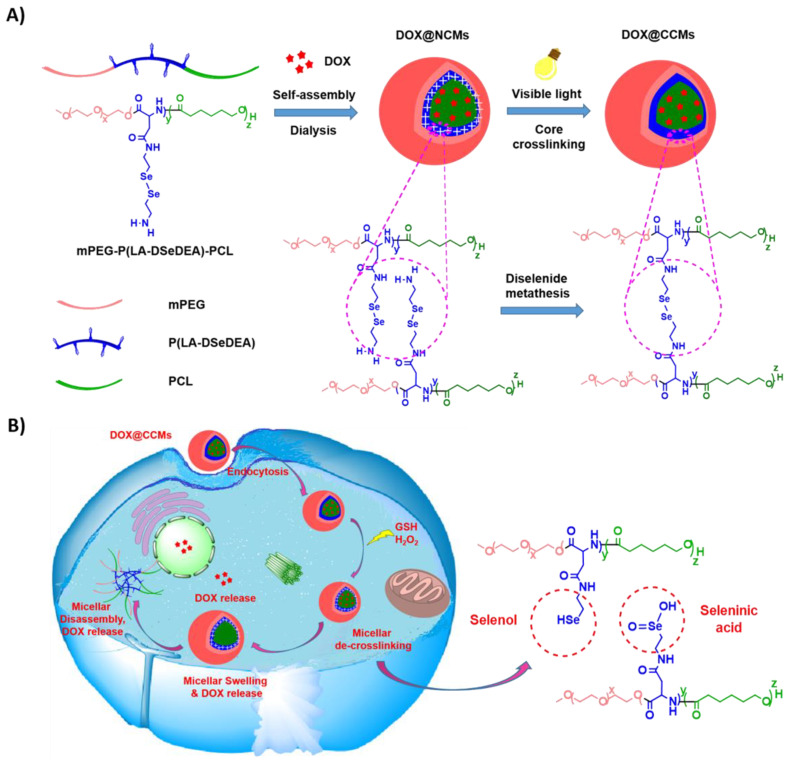
(**A**) Preparation of doxorubicin (DOX)-loaded mPEG-P(LA-DSeDEA)-PCL micelles and (**B**) mechanism of redox-responsive intracellular drug release from DOX@CCMs (DOX-loaded core crosslinked micelles).

DOX@NCMs and DOX@CCMs were prepared by a similar method except for a few modifications [21]. In brief, 1 mg of doxorubicin hydrochloride (DOX·HCl) was dissolved in 2 mL of DMSO and 5 µL TEA was added to neutralize it. On the separate vessel, 10 mg of mPEG-P(LA-DSeDEA)-PCL copolymer was dissolved in 3 mL acetonitrile and DMSO co-solvent (2:1 *v*/*v*), added to the above mixture, and probe sonicated for 2 min. Then, the mixture was added to 15 mL of water dropwise and the resulting solution was vigorously stirred overnight. The acetonitrile, DMSO and unbound DOX was removed by dialyzing (MWCO 1000 Da) against distilled water for 36 h in a dark environment to afford DOX@NCMs. In the case of DOX@CCMs, visible light from an ordinary tungsten lamp (25 W) was irradiated for the final 2 h of the dialysis process to initiate diselenide exchange reaction and thereby core crosslinking in DOX@NCMs (Scheme 2). The absorbance of DOX@NCMs and DOX@CCMs was measured by a UV-vis spectrometer at 490 nm, and the amount of DOX encapsulated in the cores of micelles was then determined based on the pre-established calibration curve of free DOX. The encapsulation efficiency (EE) and DLC of CCMs and NCMs were calculated using the following equations:

(1) $$\mathrm{EE}(\%)=\frac{\mathrm{Weight}\;\mathrm{of}\;\mathrm{DOX}\;\mathrm{in}\;\mathrm{the}\;\mathrm{micelles}}{\mathrm{Weight}\;\mathrm{of}\;\mathrm{DOX}\;\mathrm{fed}\;\mathrm{initially}}\times 100$$

(2) $$\mathrm{DLC}(\%)=\frac{\mathrm{Weight}\;\mathrm{of}\;\mathrm{DOX}\;\mathrm{in}\;\mathrm{the}\;\mathrm{micelles}\;}{\mathrm{Weight}\;\mathrm{of}\;\mathrm{DOX}\;\mathrm{loaded}\;\mathrm{micelles}\;}\times 100$$

#### 2.2.10. Redox Sensitivity and DOX-Releasing Behavior of Micelles

The redox sensitivity of PMs can be quantified by measuring *D*_h_ during swelling and disruption of micellar assemblies using DLS or by treating RhB-loaded micelles with a known concentration of reduced GSH and H_2_O_2_ and measuring the emission band at different time points [7]. In this study, the redox stimuli-responsive behavior of CCMs was investigated by treating RhB@CCMs against 0.0067 M PBS, 10 mM GSH, and 0.1% H_2_O_2_ at pH 7.4 and measuring the corresponding emission intensity of RhB at 1, 2, 3, 4, 6, 12, 24, 36, 48, and 72 h. In addition, the redox-responsiveness of blank CCMs was monitored by measuring the change in the intensity of light scattering of the micellar dispersions. For this purpose, CCMs were treated with 10 mM GSH and 0.1% H_2_O_2_, and the change in *D*_h_ or intensity of light scattering was measured by DLS. The in vitro redox-responsive drug-releasing behavior of DOX@CCMs was investigated by placing 2 mL of membrane sealed DOX-loaded micelles in separate vials containing 10 mL of PBS, 5 and 10 mM GSH in PBS, and 0.1% H_2_O_2_ in PBS and stirred continuously at 37 °C. At predetermined time intervals, 3 mL aliquots from each vial were taken and the absorbance of DOX was measured at 490 nm. Each time, 3 mL fresh media was added to maintain the volume constant. For each releasing media, triplicate measurements were conducted and the amount of DOX released was calculated at 1, 2, 3, 4, 6, 12, 24, 36, 48, and 72 h by using the free DOX calibration curve. For comparison purposes, the redox-responsive drug-releasing behavior of DOX@NCMs was also studied in the same manner as the DOX@CCMs.

#### 2.2.11. In Vitro Cytotoxicity Study 

The in vitro cytotoxicity of blank and DOX-loaded micelles was evaluated by MTT reduction assay [46,47,48] on HaCaT, MDCK, and HeLa cells in triplicate experiments. All cell lines were seeded into a 96-well plate at a density of 1 × 10^4^ cells/well in complete DMEM and maintained in a humidified incubator at 37 °C under 5% CO_2_ for 24 h. For the cell viability test, the original medium was refreshed and free DOX, DOX@NCMs, and DOX@CCMs of predetermined concentrations were added and incubated for 24 h. Next, the medium in each well was replaced with a fresh culture cell medium, and MTT reagent (5 mg/mL) was added to each well and incubated for a further 4 h. Finally, DMSO (100 µL) was added to each well and the plates were incubated at 37 °C for 25 min until all the formazan crystals were dissolved. The absorbance of reduced formazan in each well (triplicate samples) was measured at 570 nm, the cell viability was calculated as follows and the data were analyzed and expressed as mean ± standard deviation (SD).

(3) $$\mathrm{Cell}\;\mathrm{viability}\;(\%)=\frac{\mathrm{The}\;\mathrm{absorbance}\;\mathrm{of}\;\mathrm{test}\;\mathrm{cells}}{\mathrm{The}\;\mathrm{absorbance}\;\mathrm{of}\;\mathrm{control}\;\mathrm{cells}}\times 100$$

Similarly, the cytotoxicity of the blank mPEG-P(LA-DSeDEA)-PCL copolymer against HaCaT, MDCK, and HeLa cell lines was assessed at a concentration of 10, 20, 50, 100, 200, 400, and 600 µg/mL by following the aforementioned protocol.

#### 2.2.12. Cellular Uptake Study

The cellular uptake and intracellular localization of DOX@NCMs and DOX@CCMs were investigated by a fluorescent microscope using cancer (HeLa) and normal (HaCaT) cells. Both cell lines were grown in Dulbecco’s modified Eagle medium (DMEM) supplemented with 10% fetal bovine serum (FBS), 1% penicillin, 1% glutamine, and 1% sodium pyruvate at 37 °C and 5% CO_2_. For cellular uptake experiments, fully grown HeLa and HaCaT were trypsinized and seeded into 35 mm glass-bottom culture dishes (MatTek Corporation) at a density of 2 × 10^5^ cells/well in complete DMEM and maintained in a humidified incubator at 37 °C under 5% CO_2_ for 24 h. Then, 3 µg/mL of free DOX, DOX@NCMs, and DOX@CCMs (at equivalent DOX concentrations) were added to different treatment groups. After 3 and 9 h, cells were rinsed with PBS, stained with DAPI (300 nm) for 15 min, fixed with 4% paraformaldehyde solution, and the cellular uptake and localization of free DOX, DOX@NCMs, and DOX@CCMs were observed using a fluorescent microscope [3]. 

#### 2.2.13. Flow Cytometry Analysis

The cellular uptake and internalization of DOX@NCMs and DOX@CCMs were analyzed by a flow cytometry technique following the reported method [49]. Briefly, 5 × 10^5^ cells were seeded in six-well plates (1 mL of cell suspension per well) and allowed to grow for 24 h at 37 °C in 5% CO_2_. PBS, DOX, DOX@NCMs, and DOX@CCMs (at equivalent DOX concentration of 3 µg/mL) were added to the pre-assigned wells and incubated with cells for 9 h. After treatment, cells were washed with PBS, digested with trypsin, and centrifuged at 1000 rpm for 5 min. Then, the cells were suspended in 500 µL PBS and the cellular uptake of free DOX, DOX@NCMs, and DOX@CCMs was quantified by measuring the fluorescent intensity of DOX via the flow cytometry instrument (FACScan; Becton Dickinson, Heidelberg, Germany).

#### 2.2.14. Statistical Analysis

Results were expressed as mean ± SD of the three independent experiments. Moreover, SPSS Statistic software was used to analyze the data and differences were considered significant at *p*-value ≤ 0.05. 

## 3. Results and Discussion

### 3.1. Synthesis and Characterization of Copolymers

**Figure 1:**
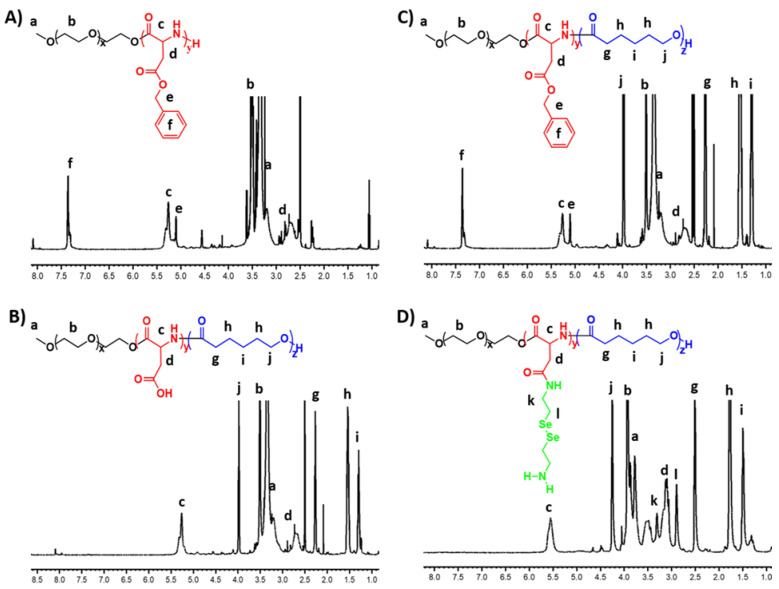
^1^H NMR spectra of mPEG-PBLA (**A**), mPEG-PBLA-PCL (**B**), and mPEG-PLA-PCL (**C**) in DMSO-d_6_; mPEG-P(LA-DSeDEA)-PCL (**D**) in CF_3_COOD.

The synthesis of the mPEG-P(LA-DSeDEA)-PCL copolymer with pendant selenocystamine moiety involves ROP reaction, hydrolysis reaction and EDC/NHS click chemistry. ROP reaction of N-carboxy anhydrides can be initiated by either mPEG-NH_2_ or mPEG-OH. Most commonly, mPEG-NH_2_ was used as a macro-initiator owing to fast reaction kinetics leading to controlled polymerization and well-defined products. However, multistep and complex synthetic procedures make the mPEG-NH_2_ macro-initiator more expensive than mPEG-OH [50,51]. In this study, we used mPEG-OH as a macro-initiator for ROP reaction of BLA-NCA in the presence of a catalytic amount of Sn(Oct)_2_ to synthesize the polypeptide mPEG-PBLA. For this purpose, we first cyclized β-benzyl-l-aspartic acid by treating it with triphosgene in THF [39]. As shown in Figure S1, the peaks at δ 2.85, 5.10, 5.24, and 7.31 ppm were assigned to –CO*CH_2_*CH, –CH_2_*CH*(CO)NH–, –O*CH_2_*C_6_H_5,_ and –*C_6_H_5_* protons, respectively, which confirmed the successful synthesis of BLA-NCA. When BLA-NCA was conjugated with the macro-initiator mPEG-OH through ROP reaction, additional resonance peaks at δ 3.25 and 3.55 ppm were observed in the ^1^H NMR spectrum of mPEG-PBLA (Figure 1A). These newly appeared peaks were ascribed to methoxyl (*CH_3_*O–) and methylene (CH_3_O*CH_2_CH_2_*O–) protons of mPEG-OH, respectively. The formation of the triblock copolymer, mPEG-PBLA-PCL was verified by the appearance of the characteristic ^1^H NMR peaks of ε-caprolactone at δ 1.33 ppm (–CH_2_*CH_2_*CH_2_–), 1.55 ppm (–COCH_2_*CH_2_*CH_2_–) and (–CH_2_CH_2_*CH_2_*CH_2_O–), 2.33 ppm (–CO*CH_2_*CH_2_–), and 3.97 ppm (–CH_2_*CH_2_*O–) beside the mPEG-PBLA peaks as depicted in Figure 1B. The disappearance of –O*CH_2_*C_6_H_5_ peaks at δ 5.10 ppm and –C_6_*H_5_* peaks at 7.31 ppm from Figure 1C asserted the successful conversion of mPEG-PBLA-PCL into mPEG-PLA-PCL through an acid-catalyzed hydrolysis reaction. The ^1^H NMR spectrum of the target compound (Figure 1D) further reaffirmed the conjugation of DSeDEA with mPEG-PLA-PCL via EDC/NHS catalyzed click chemistry. The new peaks which appeared at δ 2.92 and 3.29 ppm were due to –NH*CH_2_*CH_2_Se– and –NHCH_2_*CH_2_*Se–, respectively. Overall, the ^1^H NMR spectral data assured the synthesis of intermediates and the target copolymer, mPEG-P(LA-DSeDEA)-PCL.

**Figure 2:**
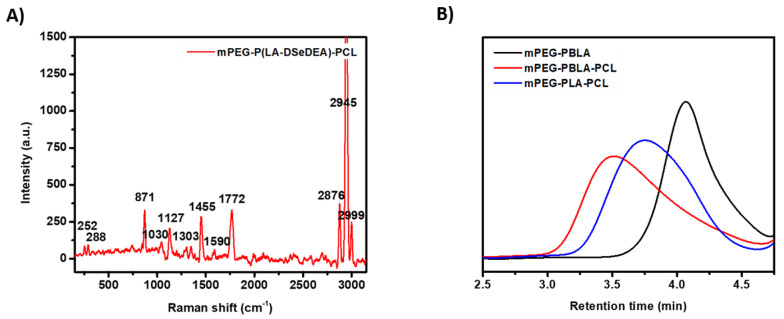
Raman shift of mPEG-P(LA-DSeDEA)-PCL (**A**) and APC traces of block copolymers (**B**).

Furthermore, the presence of characteristic functional groups of intermediates and target compounds validated the success of the synthetic procedures we followed. For instance, the presence of bands at 1720, 2942, and 2890 cm^−1^ responsible for intense C=O stretching vibration, aromatic C–H stretching vibration, and alkyl C–H vibration, respectively, confirmed the synthesis of mPEG-PBLA and mPEG-PBLA-PCL. The aromatic C=C stretching vibration at 1466 cm^−1^ and strong C–O–C stretching at 1100 cm^−1^ averred (Figure S2A,B) the successful ROP reactions which led to the formation of mPEG-PBLA and mPEG-PBLA-PCL. The aromatic C=C stretching vibrations and C–H stretching bands at 1466 and 2942 cm^−1^ significantly decreased (Figure S2C) suggesting the removal of the benzyl group from mPEG-PBLA-PCL through acid-catalyzed hydrolysis reaction and formation of mPEG-PLA-PCL. Besides C=O and alkyl C-H stretching bands, new bands at 3416, 1602, and 882 cm^−1^ due to primary N–H stretching vibration, amide and–CH_2_ groups adjacent to the Se–Se bond (Figure S2D) further asserted the conjugation of DSeDEA through click chemistry. The peaks at 252 and 288 cm^−1^ in the Raman shift of mPEG-P(LA-DSeDEA)-PCL (Figure 2A) clearly showed the presence of Se–Se and Se–C bonds in the chemical structure of the copolymer. The C–O (1030 cm^−1^) and C–C (1127 cm^−1^) bond skeletal stretching vibrations, N–H (1303 and 1590 cm^-1^) vibrations, C=O (1772 cm^−1^) stretching vibrations, –CH_2_ bending (1455 cm^−1^) and stretching (2945 cm^−1^) vibrations suggested the formation of mPEG-P(LA-DSeDEA)-PCL. Moreover, ^13^C NMR spectra of mPEG-P(LA-DSeDEA)-PCL (Figure S3) further strengthened our claim. As expected, the APC traces of the copolymers (Figure 2B) witnessed longer retention time for the lower molecular weight mPEG-PBLA and shorter elution time for the relatively higher molecular weight mPEG-PBLA-PCL copolymer. The APC result was in agreement with the molecular weights of block copolymers calculated from ^1^H NMR data (Table 1). Overall, all the data presented above averred the successful synthesis of the redox-responsive mPEG-P(LA-DSeDEA)-PCL copolymer.

### 3.2. Self-Assembly and Preparation of Core Crosslinked Polymeric Micelles

**Figure 3:**
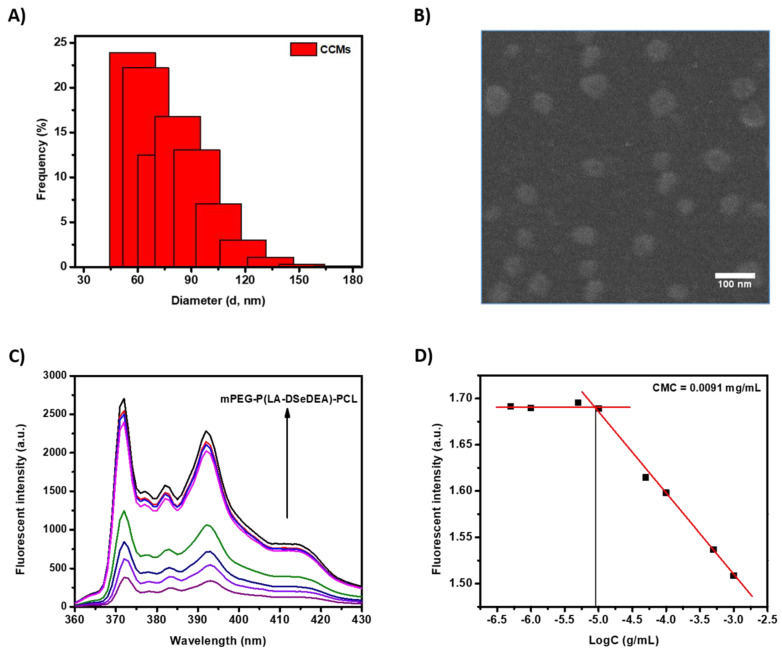
Particle size distribution of CCMs (**A**), SEM image of CCMs (**B**), fluorescent emission spectra of pyrene at different concentrations of mPEG-P(LA-DSeDEA)-PCL (**C**) and its CMC value (**D**). The scale bar in the SEM image is 100 nm.

Amphiphilic block copolymers would aggregate in aqueous solutions into PMs with a core-shell structure [23,52]. In drug delivery systems (DDS), *D*_h_ is a determinant factor governing the pharmacokinetic and pharmacodynamics properties of PMs inside the body. For instance, PMs with optimum micellar size (mostly less than 200 nm) tend to accumulate in cancer tissues through the EPR effect [53]. On the contrary, too small (less than 5 nm) or too large (submicron level) PMs suffered from rapid clearance from the body via renal excretion and the MNP system, respectively, upon intravenous administration [3,54]. In our study, we synthesized the mPEG-P(LA-DSeDEA)-PCL copolymer which self-assembled into micellar aggregates due to the presence of chemically distinct blocks, hydrophilic mPEG, and hydrophobic P(LA-DSeDEA)-PCL [3]. During the solvent exchange process, diffusion of common solvents (acetonitrile and DMSO) in an aqueous medium forces the P(LA-DSeDEA)-PCL block to undergo microphase separation and led to the formation of NCMs. When the NCMs suspension was irradiated with visible light from an ordinary tungsten lamp for 2 h, relatively stable CCMs were formed. As shown in Table 2, the CCMs showed smaller *D*_h_ (156.57 ± 4.42 nm) as compared to the NCMs (171. 55 ± 1.88 nm), which is consistent with previous reports [36,52]. The obvious *D*_h_ difference observed between NCMs and CCMs may be attributed to the intramicellar covalent Se–Se network formation and concomitant shrinkage in the interface between the hydrophobic and hydrophilic portion of the mPEG-P(LA-DSeDEA)-PCL copolymer resulting in a more compact micellar structure [27,33,34]. Both NCMs and CCMs experienced suitable ζ-potentials for cellular attachment and internalization, −9.68 ± 3.03 and −13.1 ± 1.17, respectively. According to the DLS result, monodispersed CCMs (0.23 ± 0.060) were obtained signifying the absence of notable intermicellar Se–Se bond formation (responsible for the aggregation of CCMs) during the crosslinking process (Figure 3A). Moreover, the SEM micrograph indicated that the CCMs were shown to have a spherical shape with fairly unimodal distribution (Figure 3B). The *D*_h_ of CCMs in the SEM image was around 100 nm, smaller than the DLS data owing to the difference in sample preparation procedures followed. In line with previous reports [52], an increase in the *D*_h_ of micelles was recorded when DOX was encapsulated in the hydrophobic cores of NCMs and CCMs (Figure S4). The DOX@NCMs and DOX@CCMs showed *D*_h_ values of ~188.13 ± 4.90 and ~168.14 ± 1.72 nm, respectively. As expected, the ζ-potential of DOX@NCMs and DOX@CCMs was found to be 1.55 ± 4.09 and −3.71 ± 3.51, respectively. The apparent increase in *D*_h_ and ζ-potential was associated with the insertion of DOX in the hydrophobic cores of NCMs and CCMs [3,12].

### 3.3. Critical Micelle Concentration of mPEG-P(LA-DSeDEA)-PCL

The stability of PMs at extreme dilution, such as the bloodstream, is imperative for their clinical applications [16]. CMC is the concentration of a surfactant above which unimers start to self-assemble to form a liquid colloidal solution. Above CMC, amphiphilic block copolymers spontaneously aggregate to form thermodynamically stable PMs and dissociate into unimers when diluted at concentrations below CMC. It can be estimated based on the fluorescent intensity of hydrophobic dyes such as Nile Red [20], pyrene [45], coumarin-6 [55], etc. embedded in the hydrophobic core of PMs. In this work, the CMC of mPEG-P(LA-DSeDEA)-PCL was determined through a pyrene fluorescent probe method by quantifying the emission band of pyrene when equilibrated between aqueous solution and the hydrophobic core of mPEG-P(LA-DSeDEA)-PCL at varying polymer concentrations [43,44]. As depicted in Figure 3C, the emission band intensity of pyrene was comparatively very low at lower polymer concentrations which clearly indicated that pyrene was predominantly located in a relatively polar aqueous environment since PMs were not formed to encapsulate pyrene in their hydrophobic cores. However, the emission band intensity rapidly increased with an increase in the concentration of the copolymer above the CMC as pyrene was entrapped in the nonpolar cores of a growing number of self-assembled micelles [3,54]. Figure 3D showed that the CMC of the mPEG-P(LA-DSeDEA)-PCL copolymer at room temperature was found to be 0.0091 mg/mL. The observed low CMC values indicated that nanoscale PMs could be formed at a fairly lower copolymer concentration as a result of the strong hydrophobicity of P(LA-DSeDEA)-PCL [20]. Core crosslinked mPEG-P(LA-DSeDEA)-PCL micelles would be more stable at a polymer concentration even below the CMC owing to stabilization of the micellar interface through covalent Se–Se bonds, which could help the CCMs to withstand the compressive, tensile, and shear stress forces in vivo and thereby prevent premature release of payloads upon intravenous administration [17,23].

### 3.4. Colloidal Stability of Blank and DOX-Loaded Micelles

The thermodynamic stability of PMs in physiological conditions such as pH, body fluids, etc. is of particular importance [56]. For instance, the interaction between PMs and blood components such as proteins and lipids destabilize micelles to facilitate nonselective payload release [57]. In this study, the interaction of PMs with bovine serum albumin (BSA) albumin was assessed by measuring the change in the *D*_h_ using DLS (*n* = 3). As depicted in Figure S5A, the *D*_h_ of CCMs was not significantly changed over seven days (160.08 ± 4.25) when incubated with BSA, indicating that CCMs were not affected by the possible interaction with BSA. A similar trend was noticed when DOX@CCMs were treated with BSA (171.78 ± 2.21) (Figure S5B). The presence of mPEG shell may prevent the adsorption and interaction of protein on the surface of the micelles [15,38]. Moreover, the presence of core crosslinking makes the micellar aggregate resistant to BSA triggered micellar disassembly [53]. On the contrary, NCMs and DOX@NCMs were slightly affected by BSA especially starting from the fourth day (Figure S5A,B). The lack of core crosslinking may have contributed to BSA directed minor micellar swelling. Furthermore, the stability of NCMs and CCMs was investigated by dissolving micelles with 10-fold DMF and measuring the *D*_h_ of the micelles using DLS. When a nonselective solvent, DMF, was added into NCMs, no notable light scattering signal was detected by the DLS instrument due to the complete disruption of the micellar aggregate to give unimers. On the other hand, CCMs showed an increase in *D*_h_ of the micellar structure from 156.57 ± 4.42 to 464.55 ± 3.15 nm (Figure S5C) which may be ascribed to the dissolution and swelling of the crosslinked cores by DMF. Consistent with previous reports, CCMs retained their core-shell architecture in the presence of 10-fold DMF as compared to their precursors (NCMs) [12,15]. These scenarios further confirmed the success of the core crosslinking and the extra stability of CCMs under harsh conditions [27].

### 3.5. Redox Sensitivity of mPEG-P(LA-DSeDEA)-PCL Micelles

**Figure 4:**
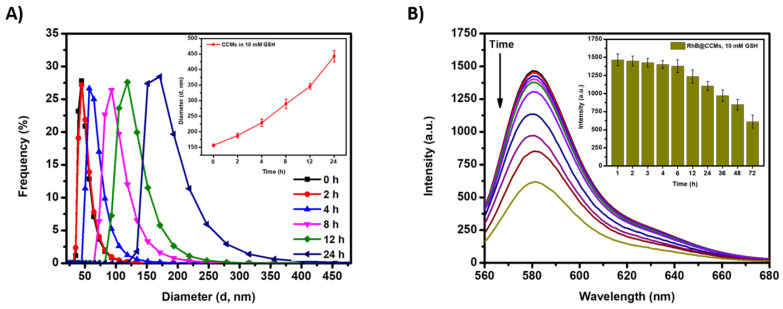
Particle size distribution of CCMs treated with 10 mM GSH (**A**); fluorescent emission spectra of RhB@CCMs treated with 10 mM GSH (**B**) for 72 h (*n* = 3). The inset in image **A** refers to the time dependent change in *D*_h_ and the inset in image **B** shows quantitative fluorescent detraction of RhB@CCMs in the presence of 10 mM GSH.

Dynamic covalent Se–Se bonds are shown to have sensitivity towards mild concentrations of reduced GSH and H_2_O_2_ [2,27]. The redox sensitivity of PMs can be quantified by measuring *D*_h_ during swelling and disruption of micellar assemblies using the DLS technique (Figure 4A). In addition, it can also be estimated by loading fluorescent dyes such as pyrene, RhB, NR, etc., and measuring the emission band at different time points after incubation with a known concentration of reduced GSH and H_2_O_2_ [7]. The fluorescent intensity of such dyes is significantly higher when solubilized and encapsulated in the nonpolar micellar cores. When dyes are released from the hydrophobic cores of PMs and exposed to a polar aqueous environment, their fluorescent intensity decreases gradually [36,54]. In this study, the redox stimuli-responsive behavior of CCMs was investigated by treating RhB@CCMs against 0.0067 M PBS, 10 mM GSH, and 0.1% H_2_O_2_ at pH 7.4. At normal physiological conditions (0.0067 M PBS, pH = 7.4), the emission fluorescence intensity band was almost the same throughout the experiment (Figure S6A), suggesting that the stable CCMs tightly restricted RhB in their core. As can be seen from Figure 4B and Figure S6B, the fluorescent emission band of RhB@CCMs decreased in a time-dependent manner when treated with 10 mM GSH and 0.1% H_2_O_2_, respectively for 72 h. Initially, there was no significant change in the intensity band of RhB but pronounced RhB release and concomitant fluorescent intensity detraction were observed in a time-dependent manner. This may be associated with the GSH and H_2_O_2_ triggered Se–Se bond cleavage and subsequent swelling and/or disintegration of the micellar assembly [20] and release of RhB into the surrounding aqueous environment to form non-fluorescent dimers [52]. Furthermore, CCMs (0.5 mg/mL of micellar solutions) were incubated in 10 mM GSH and 0.1% H_2_O_2_ and the change in *D*_h_ was measured at a different time interval. The result asserted a time-dependent increase in the micellar size in both the 10 mM GSH and 0.1% H_2_O_2_ treated groups, as depicted in Figure 4A and Figure S6C, respectively [7] owing to the redox responsive cleavage of Se–Se bonds at the interface between the hydrophobic core and hydrophilic corona of CCMs [23].

### 3.6. Drug-Loading and Releasing Behavior of Micelles

Micellar aggregates assembled from amphiphilic block copolymers are capable of solubilizing and encapsulating hydrophobic CAs such as DOX in their core [12,58]. The PMs should encapsulate a sufficient amount of CAs agents to elicit the intended anticancer activity in the region of interest. PMs with strong hydrophobic cores have shown a relatively better DLC and EE because of the favorable interaction between CAs and hydrophobic micellar cores [54]. In this study, the NCMs showed higher DLC (9.08%) and EE (50.82%) as compared to CCMs (7.31% and 42.73%, respectively) (Figure S7). Obviously, the relatively lower DLC and EE of CCMs might be ascribed to diselenide exchange prompted crosslinking and subsequent core shrinkage and efflux of DOX. Recently, DCBs have been incorporated in supramolecular chemistry and smart material fabrication [23,34,42]. Interestingly, dynamic Se–Se bonds can undergo exchange or metathesis reactions to form new Se–Se, Se–S and, Se–Te bonds when subjected to light stimuli of different wavelengths [59,60,61]. Se–Se bonds can also be cleaved by very mild redox signals such as H_2_O_2_ and reduced GSH to form seleninic acid and selenol derivatives, respectively [62]. The observed redox stimuli-responsive propensity was primarily associated with the weak bond energy of the Se–Se bond at 172 kJ/mol [25,34,63]. This endowed diselenide containing polymers to be used in the fabrication of redox stimuli-responsive CCMs for the delivery and controlled release of CAs in tumor tissue. 

**Figure 5:**
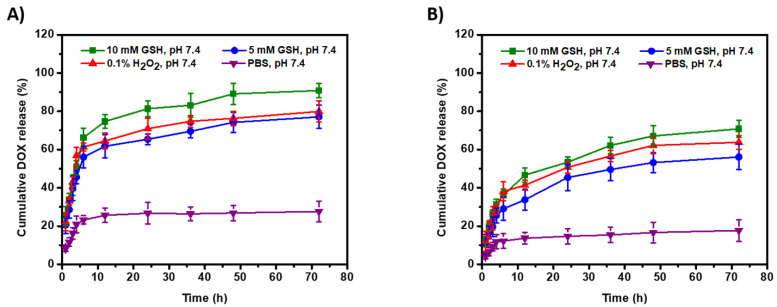
In vitro redox-responsive DOX releasing profile of DOX@NCMs (**A**) and DOX@CCMs (**B**) at 37 °C (*n* = 3).

To investigate the redox stimuli-responsive drug-releasing behavior of DOX@NCMs and DOX@CCMs, we used 5 and 10 mM GSH in 0.0067 mM PBS (pH 7.4) as a reducing stimulus, 0.1% H_2_O_2_ in 0.0067 M PBS (pH 7.4) as an oxidizing stimulus, and 0.0067 mM PBS (pH 7.4) as a representative for the normal physiological environment. As shown in Figure 5A, DOX@NCMs responded to the redox stimuli and approximately 50.46% to 66.23% of the encapsulated DOX was released within the initial 6 h. The conversion of Se–Se bonds into hydrophilic –SeOOH and –SeH in the interface accelerated the micellar disruption and release of DOX from the hydrophobic portion of DOX@NCMs and the DOX release proceeded in a sustained manner until 72 h [3]. In contrast, DOX@CCMs showed a slow DOX releasing trend in the first 6 h of the experiment when subjected to reductive GSH and oxidative H_2_O_2_ stimuli (Figure 5B). For instance, in the 6 h interval, only 29.03%, 36.25%, and 38.13% of the encapsulated DOX were released when DOX@CCMs were exposed to 5 mM GSH, 10 mM GSH, and 0.1% H_2_O_2_, respectively. The crosslinking of the micellar interface through covalent Se–Se bonds not only enhanced the serum stability of DOX@CCMs but also inhibited the burst release of DOX from their cores [27,64]. The amount of DOX released was increased gradually in a time-dependent manner and nearly 53.18%, 62.14%, and 56.11% of DOX were released within 36 h from 5 mM GSH, 10 mM GSH, and 0.1% H_2_O_2_ treated groups. The redox-responsive DOX release was extended in a sustained manner till the 72 h mark. As discussed above, the redox triggered cleavage of Se–Se bonds in DOX@NCMs and DOX@CCMs resulted in the formation of hydrophilic –SeOOH and –SeH moieties which initiated phase change in the interface between the hydrophilic and hydrophobic portions of the micelles. This phenomenon led to micellar swelling and disruption and spontaneous release of DOX from the cores of DOX-loaded mPEG-P(LA-DSeDEA)-PCL micelles. Overall, the in situ core crosslinking strategy suppressed potential DOX burst release encountered by most PMs, and the CCMs would serve as injectable DDS for the in vivo delivery and controlled release of CAs in tumor tissue.

### 3.7. In Vitro Cytotoxicity and Anticancer Effect

**Figure 6:**
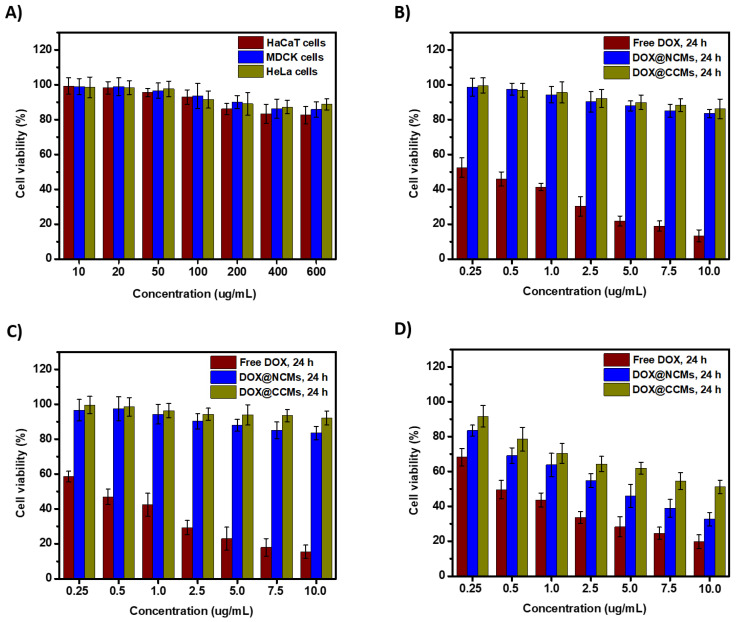
Cytotoxicity of mPEG-P(LA-DSeDEA)-PCL in HaCaT, MDCK, and HeLa cells (**A**); cell viability of free DOX, DOX@NCMs, and DOX@CCMs against HaCaT (**B**), MDCK (**C**), and HeLa (**D**) cells after 24 h (*n* = 3). The concentration of DOX@CCMs and DOX@NCMs refers to the equivalent free DOX concentration encapsulated in the micelles.

The cytocompatibility of constituent blocks and the copolymer as a whole, which form PMs, is a prerequisite for DDAs. The block copolymers could be non-toxic to normal cells at relatively fair concentrations. Taking this into account, the in vitro cytotoxicity of the blank mPEG-P(LA-DSeDEA)-PCL copolymer against normal (MDCK and HaCaT) and cancer (HeLa) cells was investigated by MTT dye reduction assay at 10, 25, 50, 100, 200, 400, and 600 mg/mL concentrations. As illustrated in Figure 6A, 82.59%, 85.93%, and 88.83% of HaCaT, MDCK, and HeLa cells, respectively, were viable when incubated with the blank mPEG-P(LA-DSeDEA)-PCL copolymer for 24 h at a maximum dose of 600 mg/mL. All cell lines showed substantial viability and the inherent biocompatibility of the FDA approved PEG and PCL segments of mPEG-P(LA-DSeDEA)-PCL may have contributed significantly to the observed viability of normal and cancer cells [52]. The therapeutic efficacy of DOX@NCMs and DOX@CCMs against normal and cancer cells were also investigated by following the aforementioned protocol. As depicted in Figure 6B,C, both HaCaT and MDCK cells exhibited about ≥ 83.51% viability in 24 h when treated with 10 µg/mL of DOX@NCMs and DOX@CCMs. As the intracellular reduced GSH and H_2_O_2_ level of normal cells are extremely low [28], it did not cause a significant change in the intracellular DOX release between DOX@NCMs and DOX@CCMs resulting in comparable cell viabilities (*p* > 0.05). On the contrary, the proliferation of HeLa cells was significantly inhibited by both DOX@NCMs and DOX@CCMs at all tested doses (Figure 6D). DOX@NCMs treated HeLa cells manifested lower cell viabilities (32.63%) than DOX@CCMs (51.15%) at a maximum dose of 10 µg/mL in 24 h (*p* < 0.01). This significant difference emanated from the intracellular drug-releasing behavior of DOX@NCMs and DOX@CCMs. The lack of covalent crosslinking in the core of DOX@NCMs expected to cause abrupt micellar swelling/disassembly, pronounced DOX release [15], and better apoptotic effect (IC_50_ = 3.80 µg/mL) when exposed to intracellular reduced GSH and H_2_O_2_ of HeLa cells (Figure S8C). On the other hand, the covalent diselenide crosslinking in DOX@CCMs requires a relatively long period of time to cleave Se–Se bonds and release a therapeutically sufficient amount of DOX to inhibit proliferation of HeLa cells. Additionally, the cell viabilities of DOX@NCMs and DOX@CCMs on HeLa cells was short of the equivalent free DOX concentrations in 24 h (*p* < 0.01). The relatively simple and fast cellular internalization of free DOX with immediate effect may have contributed to the significant inhibition of free DOX (IC_50_ = 0.47 µg/mL). Interestingly, the cell viabilities of DOX@NCMs and DOX@CCMs were consistent with their redox-responsive drug releasing profiles.

### 3.8. Cellular Uptake and Localization Study

**Figure 7:**
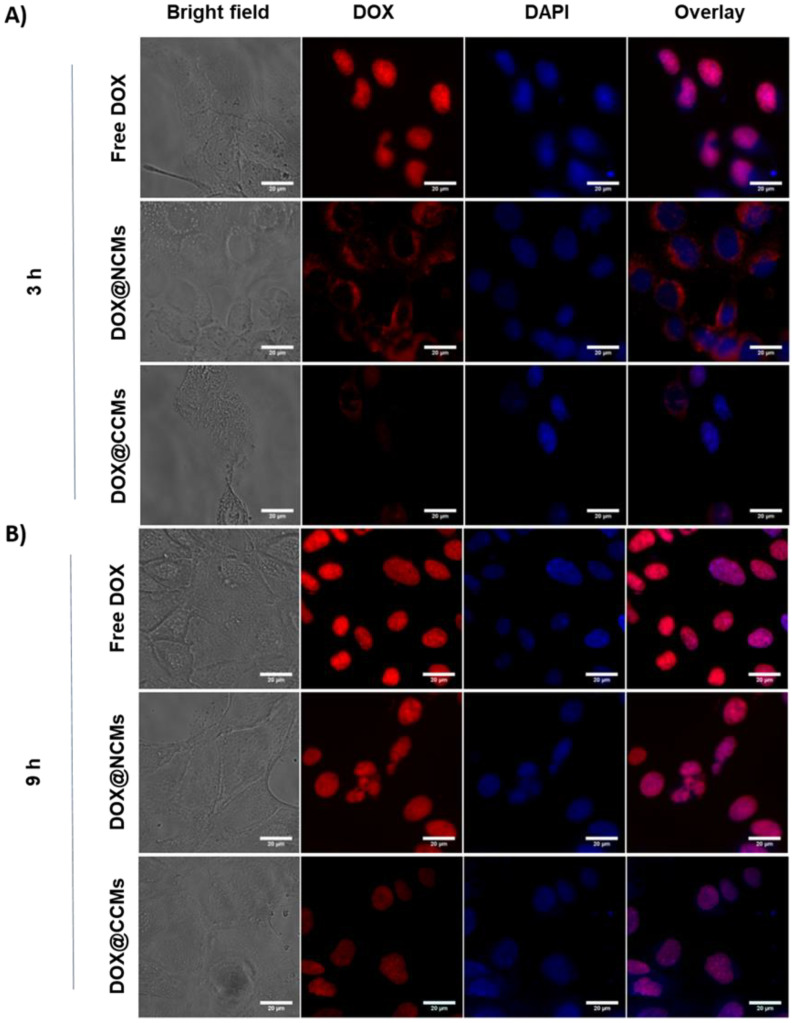
Fluorescence microscope images of HeLa cells incubated with 3 µg/mL DOX, DOX@CCMs, and DOX@NCMs for 3 h (**A**) and 9 h (**B**). The scale bar represented 20 µm.

The time-dependent passive accumulation and internalization of DOX@NCMs and DOX@CCMs were investigated by using a fluorescent microscope in HeLa and HaCaT cells, representative of cancer and normal cell lines, respectively. The inherent fluorescent intensity of DOX was used to track the cellular accumulation of NCMs and CCMs. For this purpose, 2 × 10^5^ cells (both HeLa and HaCaT) were seeded into a 35-mm-wide confocal dish and incubated (once attached to the surface) with 3 µg/mL solution of free DOX, DOX@NCMs, and DOX@CCMs. After 3 and 9 h of incubation, the cells were washed, stained with DAPI (300 nM), and fixed with freshly prepared 4% paraformaldehyde solution, and the cellular uptake and intracellular localization of free DOX, DOX@NCMs and DOX@CCMs were assessed qualitatively. As illustrated in Figure 7A, the fluorescent intensity of DOX was weak for both DOX@NCMs and DOX@CCMs at 3 h. At this time, relatively better enhancement in DOX fluorescent intensity was observed for free DOX treated HeLa cells suggesting the efficient internalization of free DOX in the cytosol of HeLa cells through passive diffusion [65]. An abrupt increase in red fluorescent intensity was noticed in the nucleus of HeLa cells treated with free DOX, DOX@NCMs, and DOX@CCMs especially at 9 h (Figure 7B). The intrinsically active and nutrition-associated endocytosis pathways of cancer cells may have contributed to the observed accumulation of DOX@NCMs and DOX@CCMs in the cytosol of HeLa cells [15]. The variation in red fluorescent intensity among the DOX@NCMs and DOX@CCMs treated groups may be due to the difference in the rate of redox-responsive cleavage of Se–Se bonds which lead to swelling/disruption and de-crosslinking (in the case of DOX@CCMs) of the micellar assemblies and release of DOX inside HeLa cells.

**Figure 8:**
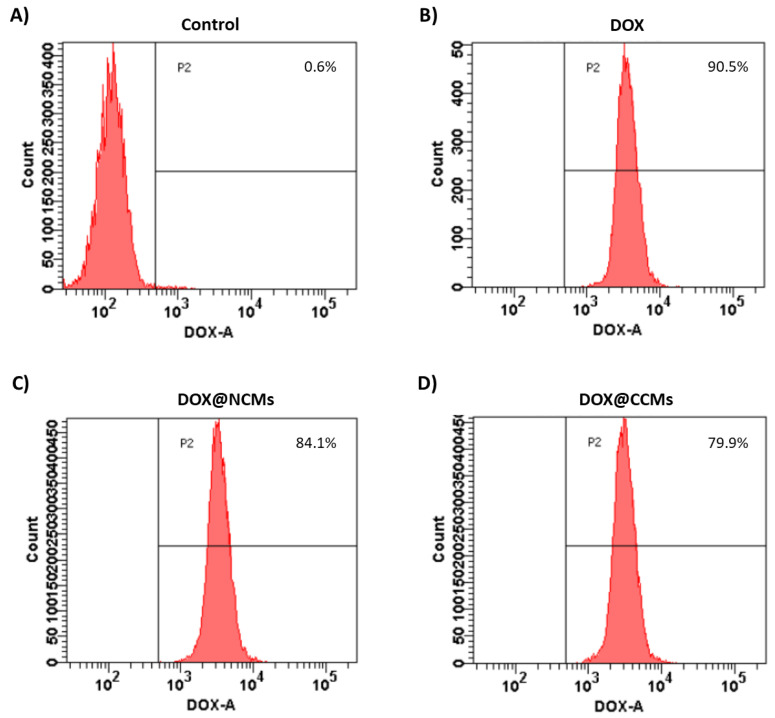
Flow cytometry analysis of HeLa cells treated with PBS (**A**), DOX (**B**), DOX@NCMs (**C**), and DOX@CCMs (**D**) for 9 h at 3 µg/mL of equivalent DOX concentration.

On the other hand, weak to mild red fluorescence was observed in the cytoplasm of HaCaT cells treated with DOX@NCMs and DOX@CCMs for 3 and 9 h, respectively (Figure S9A,B). HaCaT cells treated with free DOX demonstrated reasonable red fluorescent intensity. However, the nucleus of HaCaT cells was devoid of notable red fluorescent intensity due to the confinement of DOX in the hydrophobic cores of NCMs and CCMs. This clearly indicated that the intracellular redox signal of HaCaT cells (10-fold less than cancer cells) was not sufficient enough to trigger micellar swelling or de-crosslinking and thereby the release of DOX from the hydrophobic cores of DOX@NCMs and DOX@CCMs [3]. To further strengthen our claim, the cellular uptake and intracellular accumulation of DOX@CCMs and DOX@CCMs in HeLa cells were investigated by flow cytometry experiment. As shown in Figure 8, significant red fluorescent intensities were detected in the cytosol of HeLa cells after 9 h, asserting the efficient cellular internalization and accumulation of the DOX@NCMs (84.1%) and DOX@CCMs (79.9%) through endocytosis process. Overall, the fluorescent microscope images and flow cytometry analysis proved that mPEG-P(LA-DSeEDA)-PCL micelles, especially the extra stable CCMs are ideal platforms for the in vivo delivery and controlled release of DOX in tumor tissues. 

## 4. Conclusions

In our study, we successfully developed the mPEG-P(LA-DSeDEA)-PCL copolymer with the pendant selenocystamine group which self-assembled into micelles in aqueous solutions. Visible light-induced in situ diselenide metathesis in the interface between the hydrophobic and hydrophilic regions of NCMs resulted in the formation of CCMs. The micelles demonstrated appreciable drug loading capacities, and DOX-loaded micelles can undergo redox stimuli triggered release of DOX selectively in cancer cells, making the micelles appropriate for DDAs. Cell viability experiments on normal and cancer cells demonstrated the excellent biocompatibility of mPEG-P(LA-DSeDEA)-PCL. In addition, DOX@NCMs and DOX@CCMs exhibited comparable cellular uptake efficiency with free DOX and significantly reduced the cancer cell density in the in vitro cytotoxicity experiments. Above all, the CCMs unveiled extended colloidal stability in the presence of BSA and retained their micellar structure in the presence of 10-fold DMF, unlike their noncross-linked counterparts. These findings suggest that CCMs could be used for the in vivo delivery and controlled release of DOX in tumor tissue. Overall, we developed in situ crosslinked mPEG-P(LA-DSeDEA)-PCL aggregates which maintained their micellar architecture even at extreme dilutions; making CCMs desirable as nanocarriers for intravenous administration of payloads.

## Figures and Tables

**Table 1:** Molecular weight and polydispersity profiles of synthesized block copolymers.

Sample	Mn ^a^ (gmol^−1^)	Mn ^b^ (gmol^−1^)	Mw ^b^ (gmol^−1^)	PDI ^b^
mPEG-PBLA	8485	8396	9797	1.16
mPEG-PBLA-PCL	15,904	14,692	20,623	1.37
mPEG-PLA-PCL	13,355	11,783	14,953	1.27

^a^ The number average molecular weight was calculated from ^1^H NMR spectral data. ^b^ Determined by APC.

**Table 2:** Particle size and surface charges of blank and DOX-loaded polymeric micelles (PMs) *.

Samples	Blank PMs			DOX-Loaded PMs		
	*D*_h_ (nm)	ζ-potential	PI	*D*_h_ (nm)	ζ-potential	PI
NCMs	171.55 ± 1.88	−9.68 ± 3.03	0.19 ± 0.04	188.13 ± 4.90	1.55 ± 4.09	0.27 ± 0.03
CCMs	156.57 ± 4.42	−13.1 ± 1.17	0.23 ± 0.06	168.14 ± 1.72	−3.71 ± 3.51	0.21 ± 0.01

* The *D*_h_, ζ-potential, and polydispersities were determined by DLS (*n* = 3).

## Supplementary Materials

The following are available online at https://www.mdpi.com/1999-4923/12/6/580/s1, Figure S1: ^1^H NMR spectrum of BLA-NCA in DMSO-d_6_, Figure S2: (A) FTIR spectra of mPEG-PBLA, (B) mPEG-PBLA-PCL, (C) mPEG-PLA-PCL and (D) mPEG-P(LA-DSeDEA)-PCL, Figure S3: ^13^C NMR spectra of mPEG-P(LA-DSeDEA)-PCL in CF_3_COOD, Figure S4: (A) Particle size distribution of NCMs, (B) DOX@NCMs and (C) DOX@CCMs, Figure S5: (A) Colloidal stability of blank and (B) DOX-loaded micelles incubated with BSA; (C) micellar size distribution against 10-fold DMF, Figure S6: (A) Fluorescent emission spectra of RhB@CCMs incubated with PBS and (B) 0.1% H_2_O_2_ for 72 h; (C) redox stimuli triggered CCMs swelling in the presence of 10 mM GSH and 0.1% H_2_O_2_, Figure S7: (A) Absorbance of DOX, (B) the calibration curve for serial concentrations of free DOX; (C) the absorbance of DOX@NCMs and (D) DOX@CCMs, Figure S8: (A) IC_50_ value of DOX in HaCaT, (B) MDCK, and (C) HeLa cells treated with free DOX, DOX@NCMs, and DOX@CCMs for 24 h (n = 3), Figure S9: Fluorescence microscope images of HaCaT cells incubated with 3 µg/mL DOX, DOX@CCMs, and DOX@NCMs for 3 and 9 h. The sale bar is 20 µm.
