# Supplementary material for: Fabrication of Core Crosslinked Polymeric Micelles as Nanocarriers for Doxorubicin Delivery: Self-Assembly, In Situ Diselenide Metathesis and Redox-Responsive Drug Release
Source: Pharmaceutics. 2020 Jun 23;12(6):580. doi: 10.3390/pharmaceutics12060580 (PMC7356386; doi:10.3390/pharmaceutics12060580)
Supplement: Supplementary file 1 [file pharmaceutics-12-00580-s001.docx]

Supplementary Materials: Fabrication of Core Crosslinked Polymeric Micelles as Nanocarriers for Doxorubicin Delivery: Self-Assembly, *In Situ* Diselenide Metathesis and Redox-Responsive Drug Release

Yihenew Simegniew Birhan, Haile Fentahun Darge, Edris Yibru Hanurry, Abegaz Tizazu Andrgie, Tefera Worku Mekonnen, Hsiao-Ying Chou, Juin-Yih Lai and Hsieh-Chih Tsai


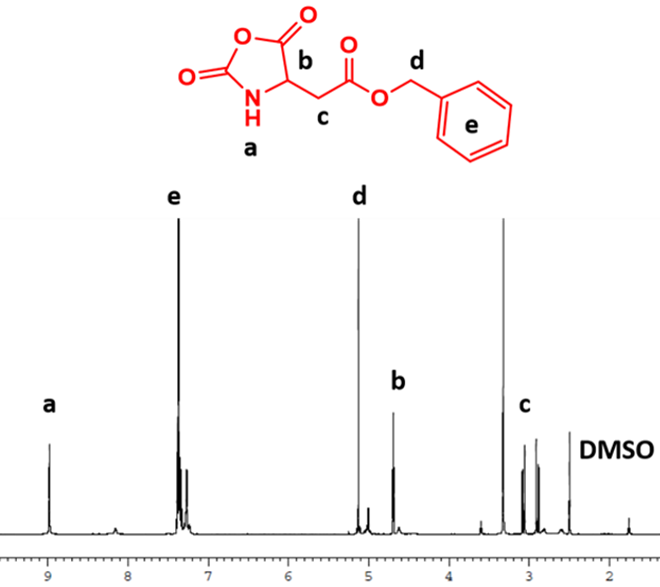


**Figure S1.** ^1^H NMR spectrum of BLA-NCA in DMSO-d_6_.


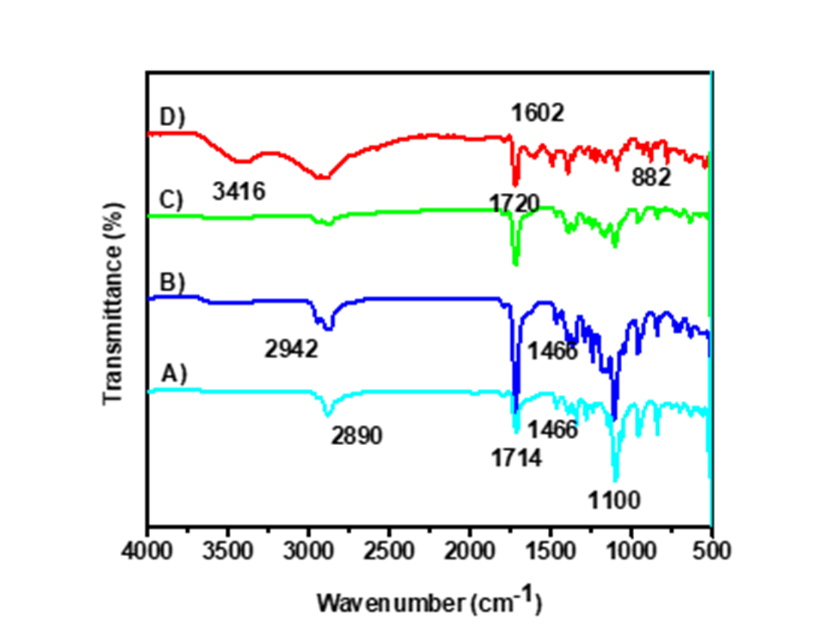


**Figure S2.** (**A**) FTIR spectra of mPEG-PBLA, (**B**) mPEG-PBLA-PCL, (**C**) mPEG-PLA-PCL and (**D**) mPEG-P(LA-DSeDEA)-PCL.


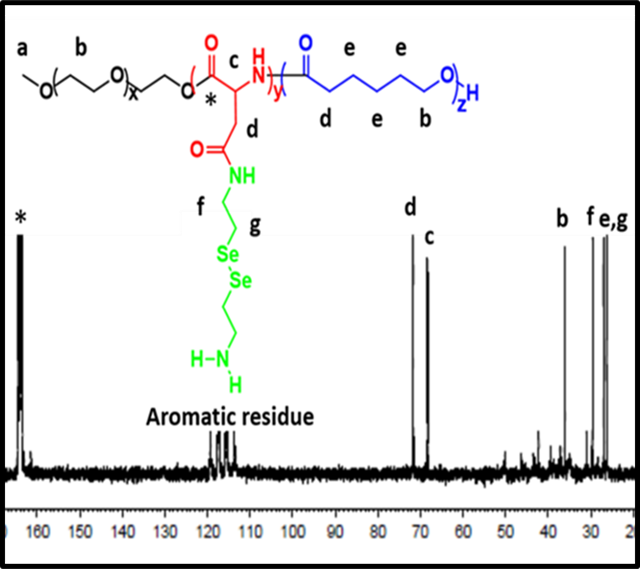


**Figure S3.** ^13^C NMR spectra of mPEG-P(LA-DSeDEA)-PCL in CF_3_COOD.


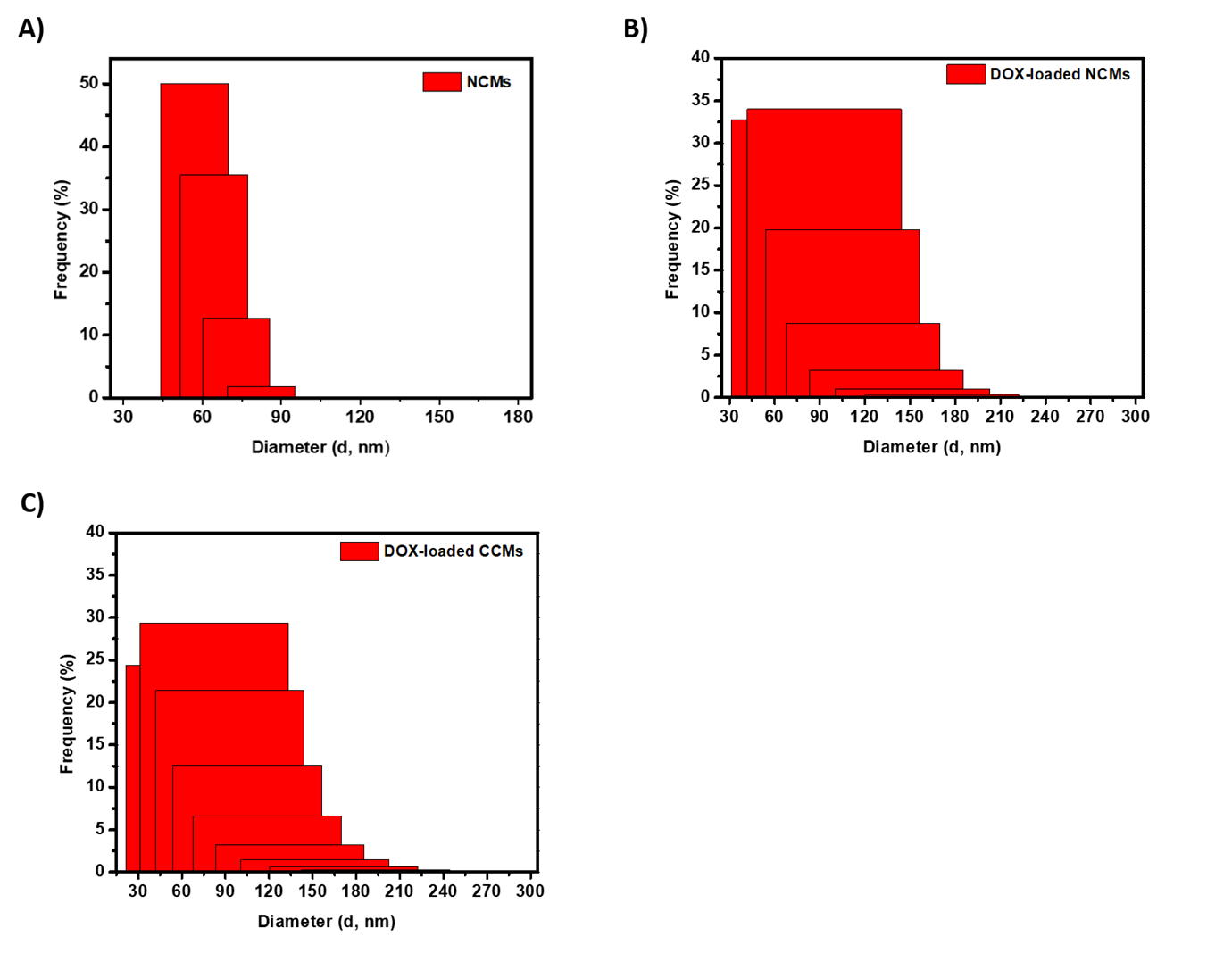


**Figure S4.** (**A**) Particle size distribution of NCMs, (**B**) DOX@NCMs and (**C**) DOX@CCMs.


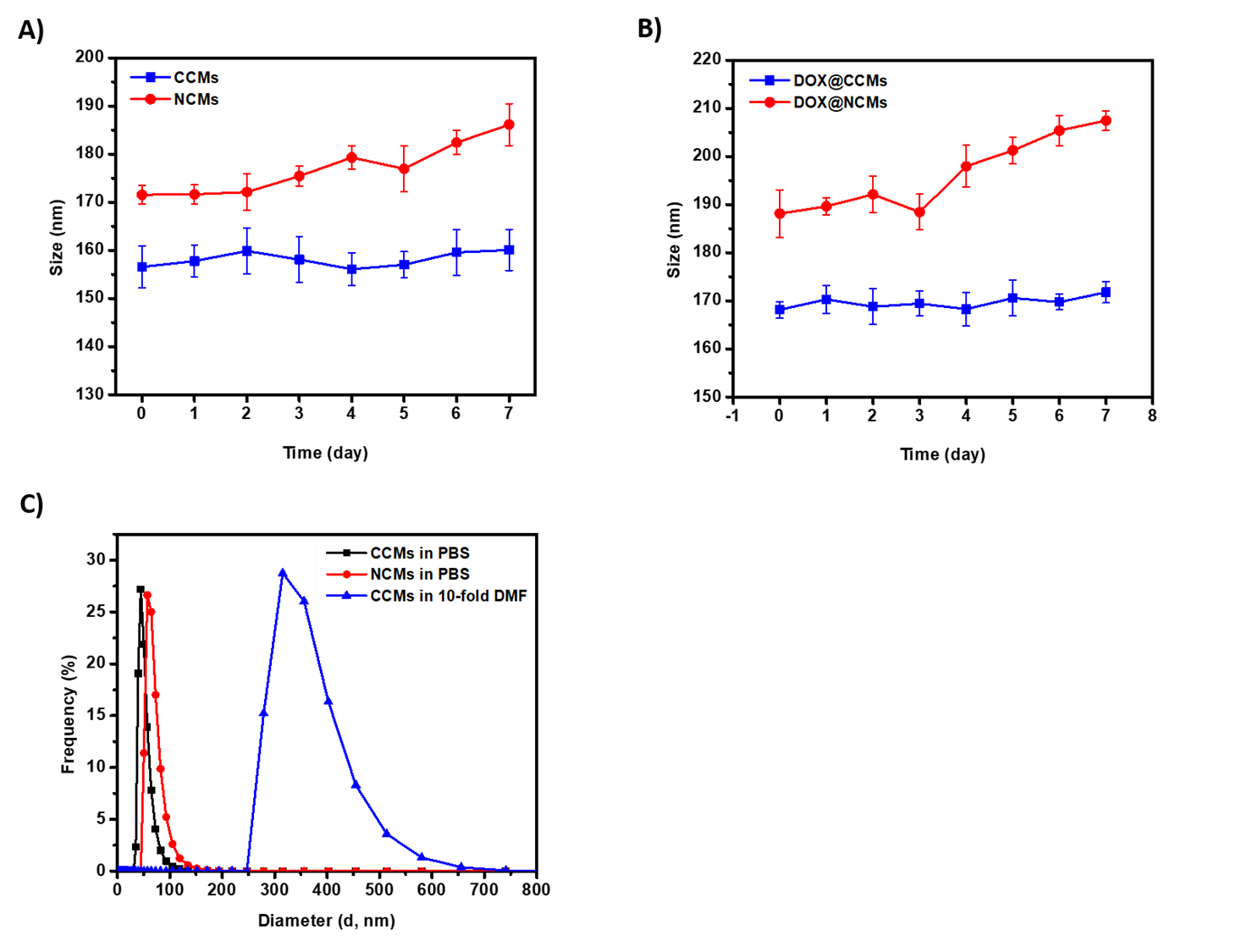


**Figure S5.** (**A**) Colloidal stability of blank and (**B**) DOX-loaded micelles incubated with BSA; (**C**) micellar size distribution against 10-fold DMF.


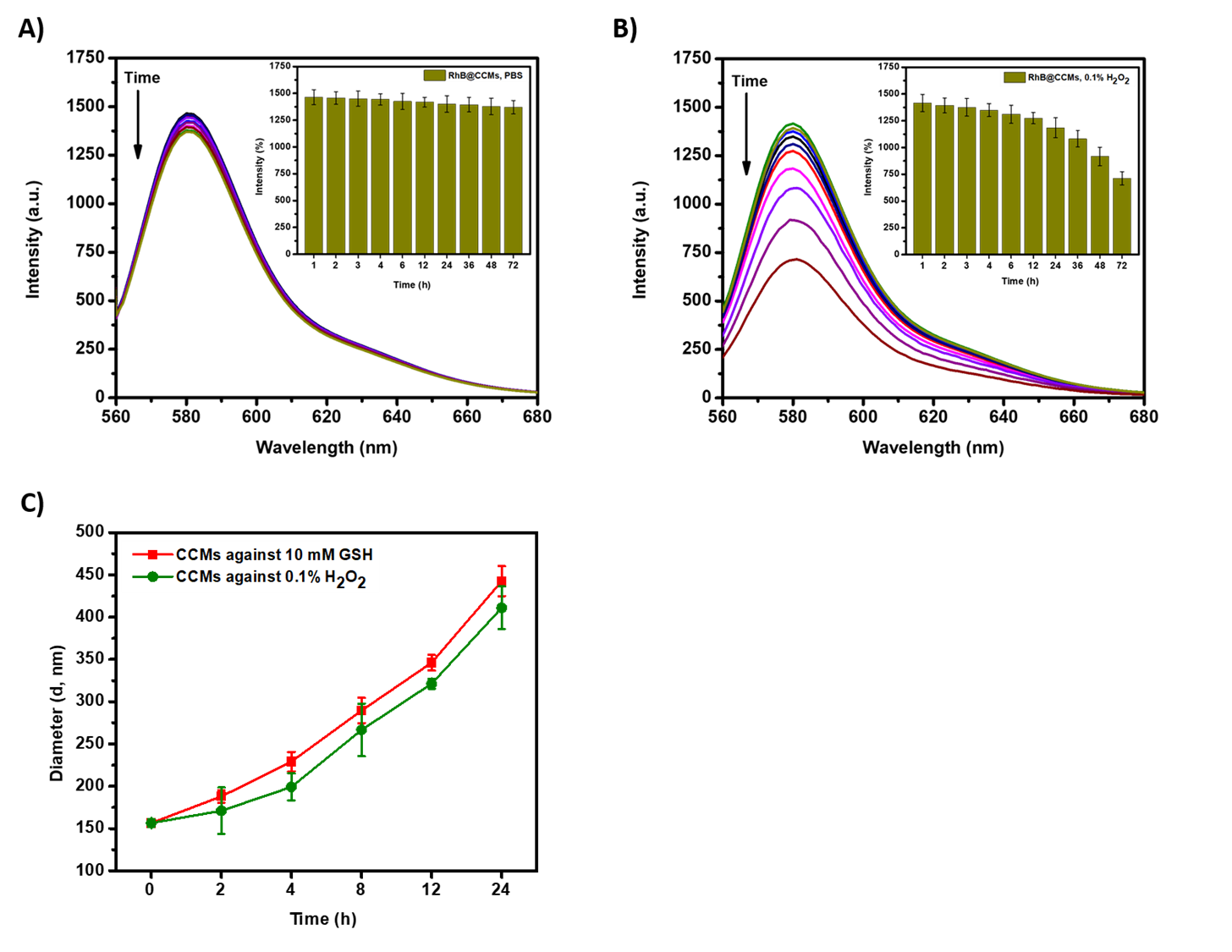


**Figure S6.** (**A**) Fluorescent emission spectra of RhB@CCMs incubated with PBS and (**B**) 0.1% H_2_O_2_ for 72 h; (**C**) redox stimuli triggered CCMs swelling in the presence of 10 mM GSH and 0.1% H_2_O_2_.


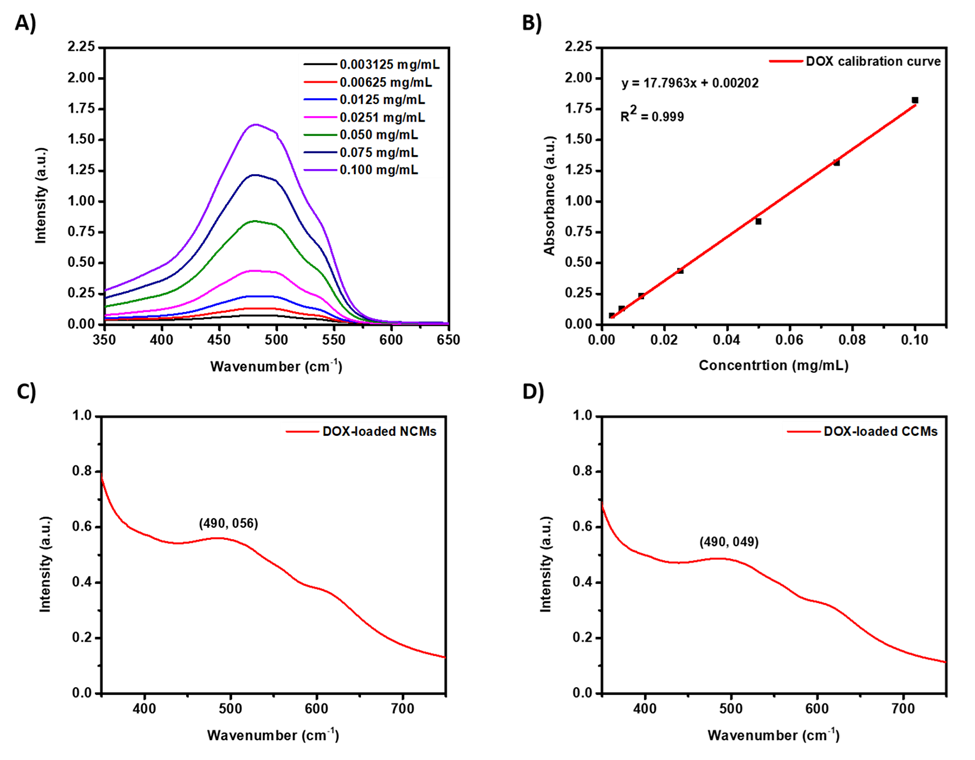


**Figure S7.** (**A**) Absorbance of DOX, (**B**) the calibration curve for serial concentrations of free DOX; (**C**) the absorbance of DOX@NCMs and (**D**) DOX@CCMs.


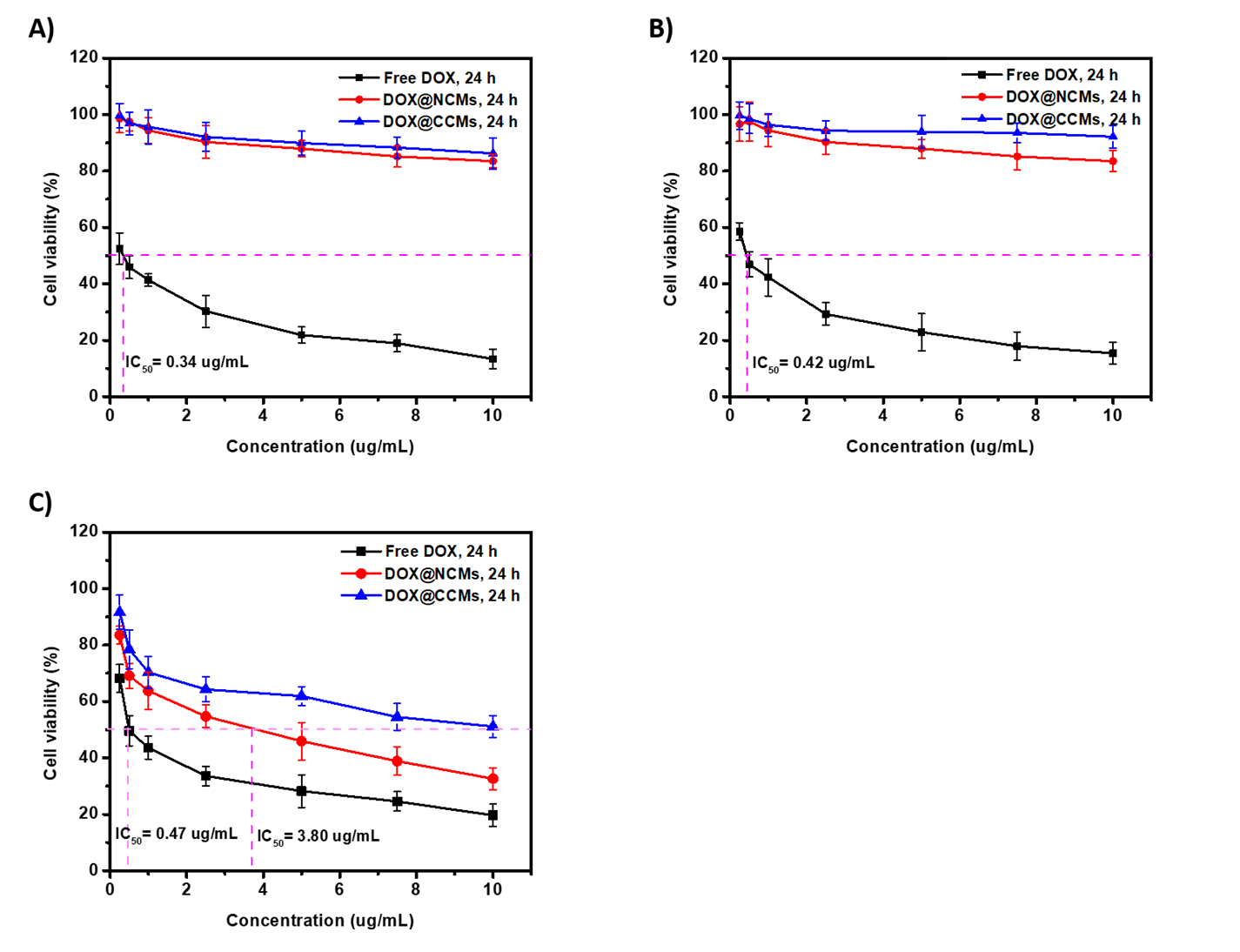


**Figure S8.** (**A**) IC_50_ value of DOX in HaCaT, (**B**) MDCK and (**C**) HeLa cells treated with free DOX, DOX@NCMs and DOX@CCMs for 24 h (*n*=3).


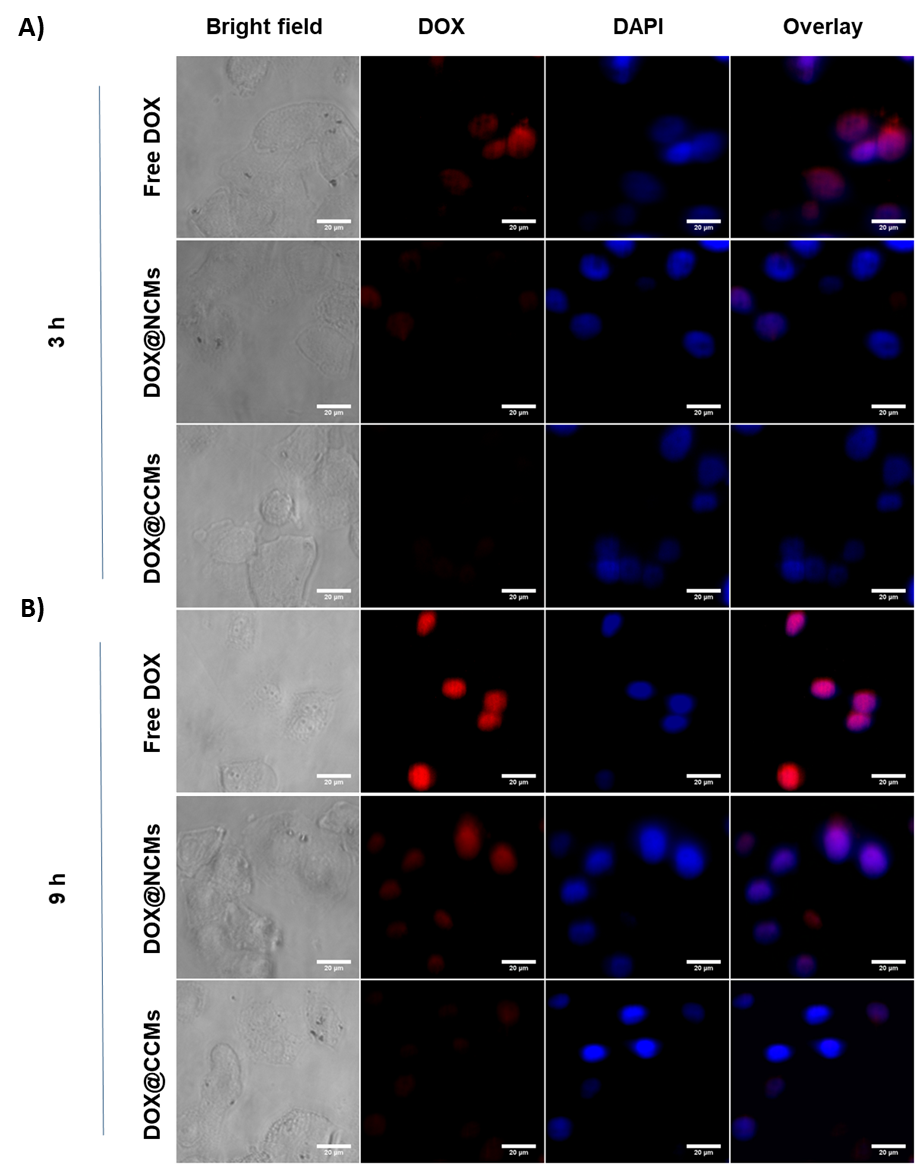


**Figure S9.** Fluorescence microscope images of HaCaT cells incubated with 3 µg/mL DOX, DOX@CCMs, and DOX@NCMs for 3 h (**A**) and 9 h (**B**). The sale bar is 20 µm.
